# The effect of 6 months of structured strength or endurance exercise program on weight loss after gastric bypass surgery in a randomized controlled trial

**DOI:** 10.1007/s00423-025-03731-7

**Published:** 2025-06-14

**Authors:** Stefanie Lehmann, Undine Gabriele Lange, Andreas Oberbach, Ulf Retschlag, Roland Morgenroth, Harald Busse, Christiane Prettin, David Petroff, Lena Seidemann, Matthias Blüher, Arne Dietrich

**Affiliations:** 1 Integrated Research and Treatment Center (IFB) Adiposity Diseases, Leipzig University Medical Center, Leipzig, Germany; 2 Institute of Clinical Immunology, University of Leipzig, Leipzig, Germany; 3 Department of Diagnostics, Fraunhofer Institute for Cell Therapy and Immunology, Leipzig, Germany; 4 Medical Department III – Endocrinology, University of Leipzig, NephrologyLeipzig, Rheumatology Germany; 5 Department of Cardiac Surgery, Ludwig-Maximilians-University, Munich, Germany; 6 Department of Diagnostic and Interventional Radiology, Leipzig University Hospital, Leipzig, Germany; 7 Clinical Trial Centre, University of Leipzig, Leipzig, Germany; 8 Helmholtz Institute for Metabolic, Obesity and Vascular Research (HI-MAG) of the Helmholtz Zentrum München at the University of Leipzig and University Hospital Leipzig, Leipzig, Germany; 9 Department of Bariatric, Metabolic and Endocrine Surgery, Clinic for Visceral, Transplant,, Thoracic and Vascular Surgery, University of Leipzig Medical Center, Leipzig, Germany

**Keywords:** Postoperative weight loss, Structured exercise program, Strength training, Endurance training, Bariatric surgery

## Abstract

**Purpose:**

To examine the effects of differently structured exercise programs (strength training (ST) vs endurance training (ET) vs a control group (CG)) on glucose metabolism and weight loss following Roux-en-Y Gastric Bypass (RYGB).

**Methods:**

After RYGB, patients were randomized to a standardized ST or ET program or a control group, the intervention started within 28 days. Outcomes at 6 months were glucose and lipid metabolism, anthropometrics, inflammation, and quality of life.

**Results:**

93 patients were randomized (30 in ST and 31 in ET, 32 in CG; 28% with type 2 diabetes mellitus, 8.5% insulin-dependent). Total weight was − 2.5 kg (95% CI − 4.7 to − 0.4, p = 0.023) lower in pooled intervention group (PIG) and fat mass according to bioelectrical impedance analysis was − 3.0 kg (95% CI − 5.0 to − 1.0, p = 0.0037) lower in the PIG. Fat-free mass decreased by − 4.2 kg with no difference between the groups. The primary endpoint, glucose concentration after a 2 h oral glucose tolerance test, did not differ between the PIG and the CG, − 0.29 mmol/L (95% CI − 1.22 to 0.63, p = 0.54). Similarly, we did not detect any differences in lipid metabolism, inflammation, and quality of life between the groups.

**Conclusion:**

In our study, we found that an additional exercise training 6 months postoperatvely- independent of the type of training- resulted in greater weight loss and loss of fat mass. However, it had no effect on glucose/lipid parameters or inflammation beyond the surgery itself.

## Introduction

Overweight and obesity are global health challenges of the twenty-first century and, in addition to high patient morbidity and mortality, are associated with a heavy financial burden on health care systems. The superiority of bariatric surgery in the effectiveness of treatment compared to conservative therapy is well-known [1–3]. The classic **R**oux-en-**Y G**astric **B**ypass (RYGB) has been shown to be a very effective and long-lasting procedure in the treatment of obesity as well as obesity-associated comorbidities such as type 2 diabetes mellitus (T2D) [4]. However, despite good diabetes remission rates of 60–70% shortly after RYGB, the relapse rates mainly after 3 to 5 years remain problematic. A recent meta-analysis by Yu et al. found a relapse rate in long-term follow-up of 30% [5]. Besides longer duration of T2D, higher preoperative HbA1c level and insulin treatment prior to surgery, less postoperative weight loss especially during the first year is a risk factors for relapse [6–8]. It may therefore be advisable to maximize weight loss in the first postoperative year.

The positive effects on metabolism after exercise programs after bariatric surgery are known, but clear exercise recommendations after bariatric surgery are still pending [9]. In recent years, some studies have suggested strong effects from strength training (ST) on glucose metabolism parameters [10–14] and inflammation parameters [15, 16]. But meta-analyses which investigated the influence of different exercise programs (endurance training (ET), ST and combined training (ET + ST)) resulted in a very heterogeneous picture regarding the superiority/inferiority of one training modality over the other [17–21]. One possible explanation is that study periods and training modalities were different and, in some cases, only small numbers of patients were involved.

For that reason, we designed a randomized controlled trial to compare post-operative effects of ST and ET on glucose metabolism parameters as well as anthropometrics, lipid metabolism, inflammation, and quality of life.

## Material and methods

### Study design

The trial was performed between April 2012 and April 2019 at our center. It was a monocentric, controlled, randomized, open-label, parallel group, prospective study regarding the influence of a postoperative exercise intervention at 6 months on glucose and lipid metabolism, weight loss, inflammation, and quality of life in bariatric surgery patients with severe obesity. Following surgery, patients were randomized to three arms: ST vs ET vs a control group (CG). The trial (Universal Trial Number (UTN): U111-1131–0765; registered 31.05.2012) was approved by the local Ethics Committee (number: 363–11-ff-07032011), and was performed in accordance with the declaration of Helsinki. All subjects gave written informed consent to use their data in pseudonymized form for research purposes before taking part in the study.

### Inclusion and exclusion criteria

Inclusion criteria were: age 18–60 years, **B**ody **M**ass **I**ndex (BMI) ≥ 40 kg/m^2^ and < 60 kg/m^2^ or BMI > 35 kg/m^2^ if obesity associated comorbidities were present. Patients were randomized after RYGB if surgery was without complications.

Exclusion criteria were: any chronic inflammatory or malignant disease, type I diabetes, contraindications for sports therapy, drug or alcohol abuse, previous bariatric surgery, untreated thyroid dysfunction, pregnancy or breast-feeding women, expected non-compliance, participation in other interventional trials except for the local IFB Baro-Diet trial [22], where the intervention was completed before surgery.

### Randomization and trial interventions

Patients were randomized to ST, ET or CG in a 1:1:1 fashion using Pocock’s minimization algorithm [23] with a stochastic component by software programmed at the clinical trial centre in Leipzig and accessed via a web interface. They were stratified by sex (female/male), age (< 45 years/≥ 45 years), BMI at screening visit (35–50 kg/m^2^/> 50–60 kg/m^2^) and type II diabetes status at screening visit (non-diabetic; diabetic without insulin treatment; diabetic with insulin treatment). The performed operation was a standardized Roux-en-Y gastric bypass with a biliopancreatic limb length of 120 cm and an alimentary limb length of 80 cm. The volume of the gastric pouch was 15–20 ml with < 2.5 cm width of the gastroenterostomy. Surgical technique and preoperative patient preparation (2-week very low calories diet) were constant over the recruitment period.

Interventions started 21 to 28 days after the operation. Supervised ET was performed on cycle ergometers, mountain climbers, cross trainers and treadmills on two non-consecutive days of the week for 65 min in total. There were an additional 10 min of warming up and cooling down. The training plan was adjusted every 8 weeks and the intensity of the training was checked at every training session to achieve training at sub-maximal level. Therefore, the subjects completed graded bicycle-ergometer test to volitional exhaustion. After a resting state of at least 3 min to measure steady state conditions, a level exercise test with an increase in work load of 20 watts per 30 s, starting with unloaded cycling plus the ergometer related permanent load was performed. Exercise tests are applied according to current guidelines for exercise testing [24].

Supervised ST was performed on training machines on two non-consecutive days of the week. The duration was again 65 min per session plus a brief warm-up period involving 10 min of moderate cycling at low intensity. Training aimed for muscle hypertrophy and consisted of three sets per muscle group. One set consisted of 8–12 repetitions, without interruption, until severe fatigue occurred. The ST program consisted of exercises for all major muscle groups. Exercises for the upper body included chest press and butterfly (M. pectoralis), shoulder press (M. deltoideus, M. trapezius), pull downs (M. latissimus dorsi), vertical row (M. trapezius) and crunches (abdominal muscles). Lower body exercises included leg press (M. quadriceps femoris), hip extensions (M. gluteus, M. biceps femoris) and leg curls (M. biceps femoris).

We chose a training intensity which is easy to standardize for both intervention groups, i.e. 70% of maximal heart rate determined in ergometry and 70% of maximal power (i.e. 8–12 repetitions of each exercise) and individual rating of perceived exertion based on an individual performance assessment. In terms of training duration, interventions initially aimed to train and maintain at least 150 min per week at moderate intensity. Our protocol with 2 training sessions per week of 65 min each (total duration 130 min/week + 20 min warm-up) corresponds to the recommendations of the American College of Sports Medicine [25] and to the postoperative recommendations of the Obesity Management Task Force of the European Association for the Study of Obesity [9].

Each training session was documented via a written protocol supervised by the local exercise trainer. These protocols were reviewed by a sports scientist every month and the exercise programs adapted accordingly.

The CG received advice to exercise twice a week for about 65 min. Patients were asked to document their exercise.

Because of the nature of the interventions, participants were aware of their assigned intervention (open-label protocol).

In addition, all participants were guided to adhere to a hypocaloric balanced diet rich in protein with an energy intake of 1200 kcal per day for women and 1400 kcal per day for men according to the European guidelines for Obesity Management in Adults [26]. Participants were provided with meal plans and instructed to self-monitor dietary patterns. The food intake was controlled at baseline, 2, 3 and 6 months via diet protocols (3 days including one weekend day). A nutritional scientist checked and evaluated these protocols.

### Measurement of outcome parameters

For the oral glucose tolerance test (oGTT) plasma glucose concentrations were measured at 2 h (2 h-oGTT). Patients underwent an oGTT at the screening visit, at the baseline visit, and after 3 and 6 months. The test was performed after an overnight fast with 75 g standardized glucose solution (Accu Chek Dextrose oral glucose test®, Roche, Mannheim, Germany). Anthropometric parameters were measured at screening visit, baseline visit, after 3 and 6 months by **B**ioelectrical **I**mpedance **A**nalysis (BIA) as previously described [22]. Laboratory parameters were taken at screening visit, baseline visit and after 1, 3 and 6 months. Plasma and serum samples were collected in the morning after overnight fasting. Concomitant diseases and medication were queried at screening visit, baseline visit and after 1, 3 and 6 months.

**I**nternational **P**hysical **A**ctivity **Q**uestionnaire (IPAQ) and the **S**hort **F**orm 36 (SF-36) health survey had to be completed at the baseline visit and after 6 months.

Assessment of nutritional intake was performed using self-reported food frequency questionnaires administered through a computer at screening, baseline, after 2, 3 and 6 months.

All study participants received an actimeter at screening, baseline, after 3 months and after 6 months to evaluate general activity. It was to be worn for 3 days, preferably including one day at the weekend and two weekdays. The number of training weeks was documented by a local exercise trainer.

### Outcomes

The primary outcome was the glucose concentration after 2 h-oGTT at 6 months. Other predefined outcomes at all available points in time were anthropometrics (weight, BMI, Waist to Hip Ratio (WHR)); fat mass and fat-free mass determined by BIA, laboratory parameter (fasting glucose and insulin, HbA1c, Homeostasis Model Assessment (HOMA-Index), cholesterol, triglycerides, High Density Lipoprotein (HDL) and Low Density Lipoprotein (LDL), leukocytes c-Reactive Protein (CRP), interleukin 6) as well as the IPAQ and the SF-36 health survey.

Excess Weight Loss (EWL) (in percent) was included as an exploratory endpoint after completion of the trial.

All parameters were determined between the respective intervention group and the control group, as well as between the pooled intervention group (PIG) and the control group.

### Sample size

A 2 h-oGTT glucose concentration of 6.2 mmol/l was expected at the start of training (28 days postoperatively) and a value of 5.3 mmol/l in the control group after 6 months, based on the following studies [27, 28]. After 6 months of endurance training, we expected the value to drop to 4.8 mmol/l. The drop was expected to be more pronounced in the strength training group with 4.4 mmol/l. A standard deviation of 0.9 (intervention) and 1.3 mmol/l (control) was expected [29]. With the conservative Mann–Whitney test with adjustments for uniform distribution, we found that 40 patients/arm for the exercise arms (i.e. 80 total) and 40 in the control arm have to be analyzed to have 90% power at the 5% significance. The drop-out rate was assumed to be 15% so that 140 patients were to be randomized. Due to slower patient accrual than anticipated, recruitment was stopped early with an anticipated power of 84%.

### Sub-Groups

No subgroup analyses were pre-specified in the study protocol. However, the statistical analysis plan foresaw a sub-group analysis done for diabetic patients. “Diabetic” is defined as those with known diabetes or those diagnosed with diabetes based on one of the following criteria being met at screening (fasting glucose ≥ 7.0 mmol/L; 2 h-OGTT plasma glucose concentration ≥ 11.1 mmol/L; HbA1c ≥ 6.5%; use of anti-diabetic medication). “Pre-diabetes” is defined by 5.6 ≤ fasting glucose < 7.0 mmol/L, 7.8 ≤ 2 h-OGTT < 11.1 mmol/L or 5.7 ≤ HbA1c < 6.5% and not on diabetes medication. A sub-group analysis was undertaken for active vs inactive patients based on (a) the number of training sessions attended or (b) the number of steps taken per day according to the actimeter, and where the 25% with the least and most training sessions/steps define the inactive and active groups.

### Statistical analyses

The intent to treat analyses consider all randomized patients, based on the group they were randomized to. Endpoints were analysed using mixed models with patient as a random term. The points in time were treated as categorical and not a continuous numerical variable. Adjustments for glucose lowering agents were not included in the models, but the stratification variables were taken as covariates. Some variables such as insulin were treated on a logarithmic scale. A linear model was used to analyse 2 h-oGTT. The dependent variable was 2 h-oGTT plasma glucose concentration at 6 months. The covariates were the stratification levels and the randomization group (exercise vs control, where “exercise” refers to both endurance and strength) and changes in glucose lowering agents based on the categories “improved”, “unchanged” and “worsened”. Missing data was accounted for with multiple imputation using 50 imputation sets using the “mice” package [30] and its default settings. The variables included for imputation were the stratification variables, all available 2 h-oGTT plasma glucose concentrations. As a sensitivity analysis, the baseline value of 2 h-oGTT plasma glucose concentration was included in the model. As a further sensitivity analysis, a linear mixed model as a repeated measurement analysis was performed using the same covariates as in the primary analysis, but including all oGTT values. A complete case analysis was also performed as a sensitivity analysis. All p-values are two-sided and a significance level of 5% is used throughout without adjustments for multiple comparisons meaning that significant results other than for the primary endpoint are hypothesis generating and not confirmatory. Analyses were performed with the software R version 4.2.0 or higher.

## Results

### Study adherence and patients characteristics

Of the 93 randomised patients, 89 completed the study: 30 in CG, 29 in ST arm and 30 in ET arm. Reasons for drop-outs were not given in the intervention groups; in the control group, two patients were dissatisfied with randomization to the control arm. The mean number of sessions attended was 39 (ST) and 40 (ET). In the ST group, 6 patients attended sessions for 1–19 weeks and 24 for at least 20 weeks. In the ET group, 4 patients attended sessions for 1–19 weeks and 26 for at least 20 weeks.

Regarding the sub-groups for active vs inactive patients based on number of steps: we obtained valid values from 81 patients at screening, 87 patients at baseline, 74 patients at 3 months and 83 patients at 6 months regarding steps recorded by actimeter. The mean number of steps/day in thousands was 4.0 (inactive) vs 12.9 (active) for a difference of 8.9 steps (95% CI 7.8 to 10.1). Regarding the sub-groups for active vs inactive patients regarding the number of training sessions: 14.5 (inactive) vs 55.2 (active) session were attended with a difference of 40.7 sessions (95% CI 33.1 to 48.3).

Table 1 shows the patients’ characteristics at baseline (if not otherwise stated).

**Table 1:** Patients characteristics

	Control group (n = 32)	Strength training group (n = 30)	Endurance training group (n = 31)
Number of females	23 (72%)	21 (70%)	21 (68%)
Age (years) ≥ 45	45.7 ± 8.4 17 (53%)	42.3 ± 10.3 14 (47%)	43.5 ± 9.2 13 (42%)
BMI (kg/m^2^) pre surgery > 50	47.7 ± 6.4 6 (19%)	47.9 ± 6.2 8 (27%)	48.5 ± 6.9 10 (32%)
BMI (kg/m^2^)	43.4 ± 6.1	43.0 ± 6.1	43.9 ± 6.6
Smokers (%)*	5/31 (16%)	8/30 (27%)	4/30 (13%)
Blood pressure systolic diastolic	121 ± 16 80 ± 9	128 ± 12 83 ± 12	131 ± 17 86 ± 12
Typ II diabetes (%) pre surgery Of those using insulin	7 (22%) 2 (29%)	6 (20%) 1 (17%)	13 (42%) 5 (38%)

Patients were randomized according to age, sex, BMI and T2DM status. Over all patients, were age: 44.8 ± 9.3 years, sex: 70% females, BMI: 43.5 ± 6.3 and T2DM status: 28%

### Anthropometrics

#### Total weight loss, BMI, excess weight loss and Waist Hip Ratio

There was a significantly higher TWL (p = 0.023) between PIG and CG of −2.5 kg (95% CI: −4.7 to −0.4 kg); but no significant difference between the two intervention groups.

Also, the BMI dropped significantly more in the PIG than in the CG by − 0.82 kg/m^2^ (95% CI: − 1.55 to − 0.10 kg/m^2^; p = 0.029); again, with no difference between the ST and ET group.

**Fig. 1:**
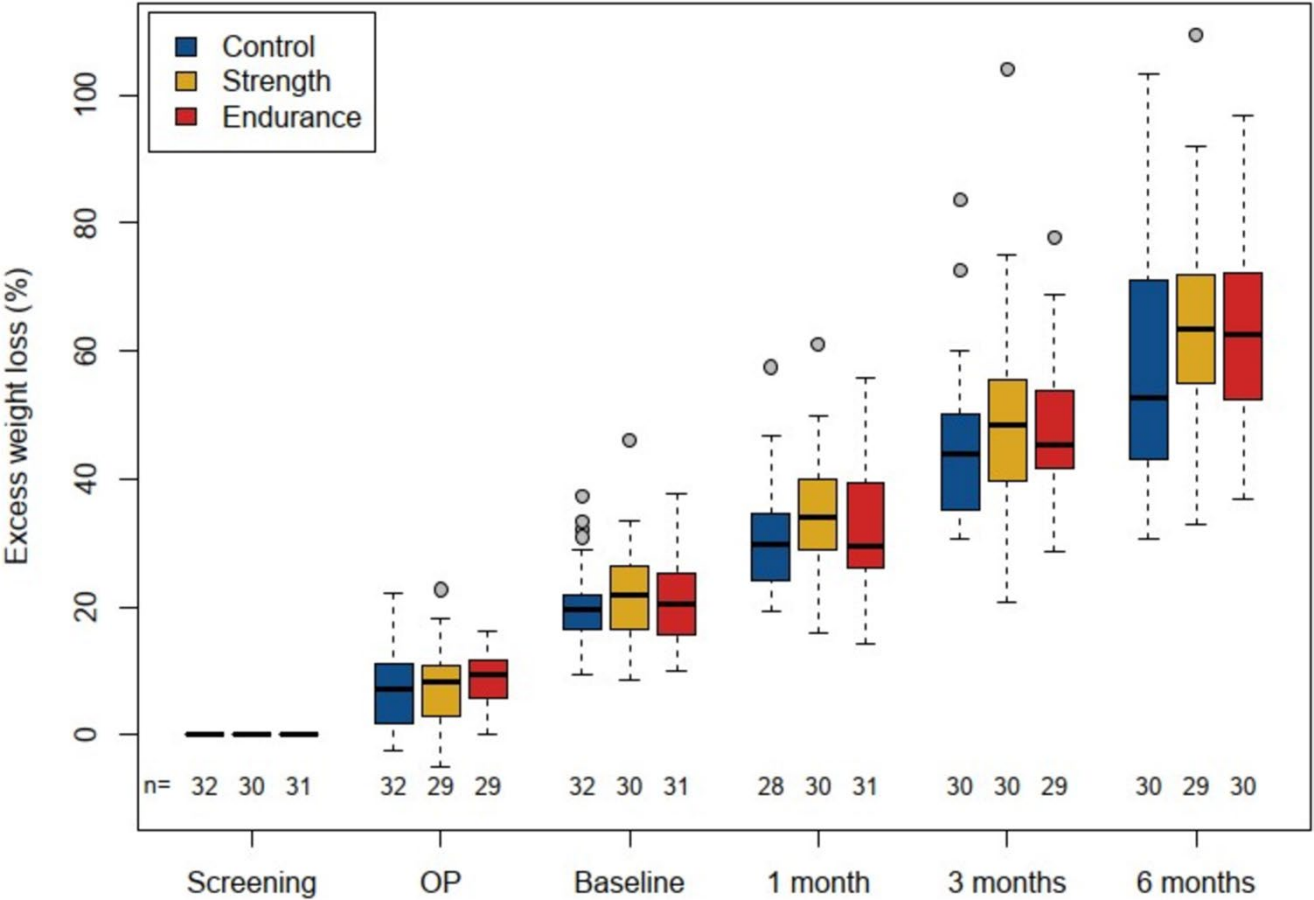
Excess weight loss over time in all groups. The excess weight loss over 6 months was significant in the PIG vs CG was + 3.47% (95% CI: 0.22 to 6.73%; p = 0.04). Excess weight loss in ST vs CG was + 3.66% (95% CI: −1.13 to 8.46%) and in ET vs CG + 3.29 (95% CI −1.5 to 8.08%), without significant difference between the intervention groups

The EWL was + 3.47% (95% CI: 0.22 to 6.73%) higher inly the PIG (p = 0.040) with no difference between the intervention groups (Fig. 1).

There were no significant differences in WHR between PIG and CG or between the intervention groups. For all anthropometrics see Table 2.

**Table 2:** Overview of all outpoints

	**6 months vs baseline**	**pooled intervention *p-*** **vs control *value***	**strength vs control**	**endurance vs control**	**strength vs endurance**
**Anthropometric** Weight (kg) BMI (kg/m^2^) Excess weight loss (%) Waist-to-hip ratio Fat mass (kg) Fat free mass (kg)	− 26.0 (− 27.9 to − 24.2) − 9.0 (− 9.6 to − 8.4) 40.4 (37.4 to 43.3) − 0.02 (− 0.19 to 0.16) − 21.9 (− 23.9 to − 19.8) − 4.2 (− 5.6 to − 2.9)	− 2.5 (− 4.7 to − 0.4) ***0.023*** − 0.82 (− 1.55 to − 0.10) ***0.029*** 3.47 (0.22 to 6.73) ***0.040*** 0.05 (− 0.06 to 0.17) *0.38* − 3.0 (− 5.0 to − 1.0) ***0.0037*** 0.2 (− 1.4 to 1.8) *0.79*	− 2.3 (− 5.5 to 0.8) − 0.76 (− 1.83 to 0.31) 3.66 (− 1.13 to 8.46) 0.09 (− 0.08 to 0.26) − 2.8 (− 5.7 to 0.1) 0.2 (− 2.1 to 2.6)	− 2.7 (− 5.9 to 0.4) − 0.89 (− 1.95 to 0.18) 3.29 (− 1.50 to 8.08) 0.02 (− 0.15 to 0.18) − 3.2 (− 6.1 to − 0.3) 0.2 (− 2.2 to 2.6)	0.4 (− 2.8 to 3.6) 0.13 (− 0.96 to 1.21) 0.37 (− 4.54 to 5.28) 0.07 (− 0.10 to 0.24) 0.4 (− 2.5 to 3.4) 0.1 (− 2.4 to 2.5)
**Laboratory** Fasting glucose (mmol/L) Fasting insulin* 2 h-oGTT (mmol/L) HOMA-IR* HbA1c (%) Cholesterol (mmol/L) HDL (mmol/L) LDL (mmol/L) TGL (mmol/L) C-reactive protein* Leucocytes (109/L) Interleukin 6*	− 0.51 (− 0.78 to − 0.24) 0.55 (0.47 to 0.65) −3.69 (−4.14 to −3.23) 0.49 (0.41 to 0.60) − 0.18 (− 0.31 to − 0.05) 0.00 (− 0.18 to 0.18) 0.34 (0.29 to 0.40) − 0.03 (− 0.18 to 0.11) − 0.53 (− 0.65 to − 0.42) 0.45 (0.33 to 0.60) 0.28 (− 0.30 to 0.87) 0.81 (0.67 to 0.99)	− 0.04 (− 0.29 to 0.21) *0.74* 0.99 (0.83 to 1.17) *0.89* − 0.29 (− 1.22 to 0.63) *0.54* 1.03 (0.85 to 1.23) *0.78* − 0.05 (− 0.20 to 0.11) *0.56* − 0.19 (− 0.40 to 0.02) *0.074* 0.03 (− 0.04 to 0.10) *0.36* − 0.17 (− 0.36 to 0.02) *0.075* − 0.10 (− 0.21 to 0.01) *0.071* 0.75 (0.56 to 1.00) *0.053* − 0.19 (− 0.62 to 0.23) *0.37* 0.92 (0.71 to 1.18) *0.51*	− 0.05 (− 0.42 to 0.31) 1.04 (0.81 to 1.33) − 0.16 (− 1.24 to 0.91) 1.08 (0.83 to 1.41) − 0.01 (− 0.24 to 0.21) − 0.24 (− 0.54 to 0.06) 0.04 (− 0.06 to 0.14) − 0.22 (− 0.50 to 0.05) − 0.13 (− 0.29 to 0.03) 0.77 (0.51 to 1.17) 0.20 (− 0.39 to 0.79) 0.98 (0.68 to 1.42)	− 0.03 (− 0.40 to 0.33) 0.94 (0.74 to 1.20) − 0.42 (−1.51 to 0.66) 0.98 (0.75 to 1.28) − 0.07 (− 0.29 to 0.14) − 0.14 (− 0.44 to 0.16) 0.03 (− 0.08 to 0.13) − 0.12 (− 0.40 to 0.15) − 0.08 (− 0.24 to 0.08) 0.73 (0.48 to 1.11) − 0.57 (− 1.15 to 0.02) 0.87 (0.61 to 1.24)	− 0.02 (− 0.39 to 0.35) 1.10 (0.86 to 1.41) 1.11 (0.85 to 1.45) 0.06 (− 0.16 to 0.28) − 0.10 (− 0.40 to 0.21) 01.2 (− 0.09 to 0.12) − 0.10 (− 0.38 to 0.18) − 0.05 (− 0.21 to 0.11) 1.05 (0.69 to 1.59) 0.76 (0.17 to 1.36) 1.13 (0.79 to 1.62)
**Life quality** **IPAQ** Energy expenditure (kcal/wk) **SF-36** Physical functions Role physical Body pain General health Vitality Social functioning Role emotional Mental health	2710 (993 to 4428) 22.7 (18.0 to 27.5) 44.1 (31.4 to 56.7) 21.6 (14.7 to 28.6) 9.2 (4.3 to 14.1) 14.7 (9.5 to 19.9) 11.0 (4.0 to 18.1) 14.3 (3.3 to 25.3) 5.1 (0.1 to 10.2)	911 (− 196 to 2019) *0.11* 0.10 (− 3.15 to 3.36) *0.95* 0.64 (− 6.58 to 7.87) *0.86* 0.51 (− 3.98 to 5.01) *0.82* − 0.07 (− 3.33 to 3.19) *0.97* 1.84 (− 1.48 to 5.17) *0.28* 2.37 (− 2.20 to 6.94) *0.31* 5.49 (− 1.65 to 12.63) *0.14* − 0.82 (− 4.30 to 2.66) *0.65*	654 (− 999 to 2307) 0.04 (− 4.78 to 4.87) − 0.28 (− 11.01 to 10.46) 0.80 (− 5.92 to 7.51) 0.25 (− 4.62 to 5.12) 2.11 (− 2.88 to 7.10) 1.46 (− 5.36 to 8.29) 4.04 (− 6.62 to 14.70) − 2.38 (− 7.51 to 2.76)	1142 (− 462 to 2747) 0.16 (− 4.64 to 4.96) 1.54 (− 9.11 to 12.19) 0.24 (− 6.38 to 6.87) − 0.37 (− 5.18 to 4.44) 1.59 (− 3.33 to 6.50) 3.24 (− 3.52 to 9.99) 6.90 (− 3.67 to 17.47) 0.68 (− 4.41 to 5.77)	− 488 (− 2156 to 1179) − 0.12 (− 5.11 to 4.87) − 1.81 (− 12.96 to 9.34) 0.55 (− 6.41 to 7.51) 0.62 (− 4.44 to 5.68) 0.53 (− 4.68 to 5.74) − 1.78 (− 8.94 to 5.39) − 2.86 (− 14.05 to 8.33) − 3.06 (− 8.42 to 2.30)

^*^Entries are n-fold changes with respect to the reference (variables treated on a logarithmic scale). All values are estimates (95% confidence interval). 2 h-oGTT = 2 h-oral Glucose Tolerance Test;IPAQ = International Physical Activity Questionnaire; SF-36 = Short form 36

#### Fat mass and fat-free mass

**Fig. 2:**
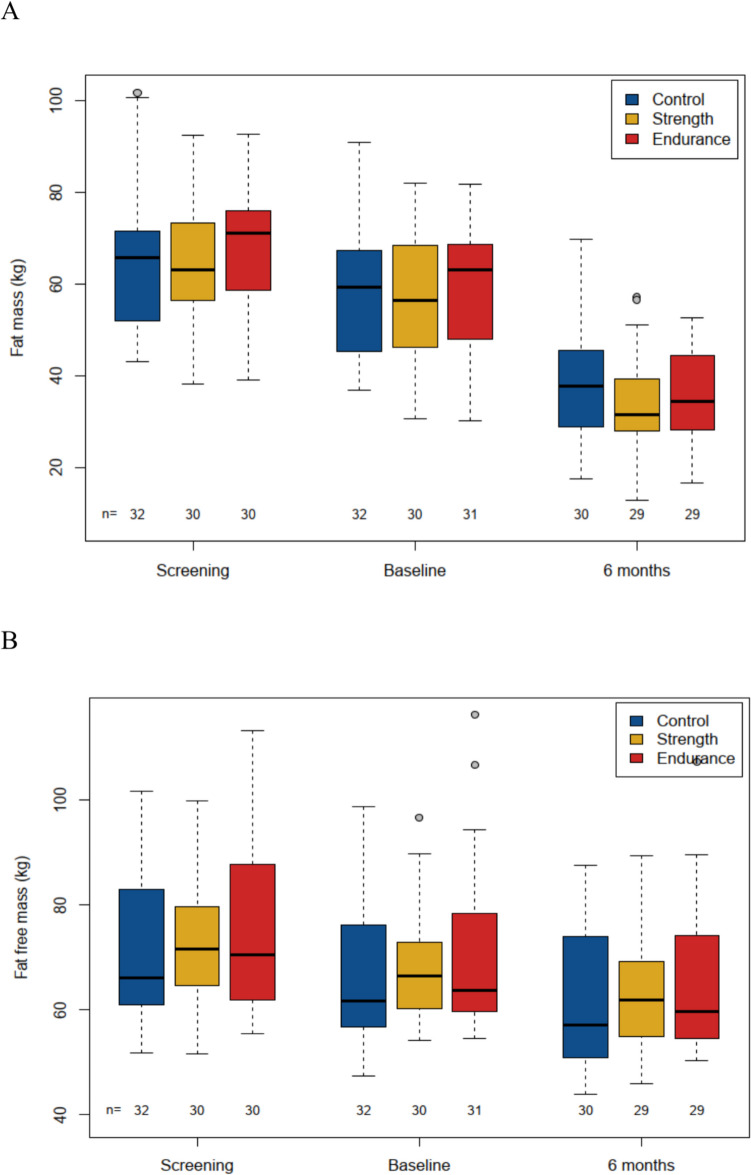
Fat mass (A) and Fat-free mass (B) over time in all groups**.** A*:* The loss of fat mass over 6 months was significant in the PIG vs CG was −3 kg (95% CI: −5 to −1 kg; p = 0.0037). Excess weight loss in ST vs CG was −2.8 kg (95% CI: −5.7 to 0.1 kg) and in ET vs CG −3.2 kg (95% CI −6.1 to −0.3 kg), without significant difference between the intervention groups. B*:* The development of fat-free mass over 6 months was in the PIG vs CG was + 0.2 kg (95% CI: −1.4 to 1.8 kg; p = 0.79). Excess weight loss in ST vs CG was 0.2 kg (95% CI: −2.1 to 2.6 kg) and in ET vs CG −0.2 kg (95% CI −2.2 to 2.6 kg), without significant difference between the intervention groups

There was a significantly lower fat mass in the PIG by − 3 kg (95% CI: − 5 to − 1 kg, p = 0.0037) as determined by BIA, but again there was no difference between the intervention groups (Fig. 2A). Fat-free mass changed very little overall (− 4.2 kg in 6 months) and we saw almost no difference between groups with + 0.2 kg (95% CI: − 1.4 to 1.8 kg; p = 0.79). The difference between ST and ET was also negligible at + 0.1 kg (95% CI: − 2.4 to 2.5), see Fig. 2B and Table 2.

### Parameters regarding glucose metabolism

The primary endpoint, 2 h-oGTT plasma glucose concentration, decreased from 8.3 mmol/L at baseline (after surgery, but before intervention) to 4.8 mmol/l at 6 months The results of the intervention group did not differ significantly, compared to the CG the ST was − 0.16 mmol/L (95% CI: − 1.24 to 0.91 mmol/L) lower and the ET − 0.42 mmol/L (95% CI: −1.51 to 0.66 mmol/L). The difference between PIG and CG was only − 0.29 mmol/L (95% CI: − 1.22 to 0.63 mmol/L; p = 0.54), where the minus sign indicates lower values in the PIG. Sensitivity analyses including the baseline value as a covariate of a mixed-model with points in time confirmed this result. Considering patients with T2D as a subgroup also showed no difference between groups. Inactive patients according to the number of steps taken had nominally higher 2 h-oGTT plasma glucose concentrations at 6 months, which were 0.96 mmol/L higher than for active patients (95% CI: − 0.26 to 2.19 mmol/L), although the 95% CI contains the value zero (p = 0.12). We were unable to detect any effect in terms of the number of training sessions attended.

There was also no significant difference in fasting plasma glucose and insulin, HbA1c and HOMA-index between the two intervention groups and CG or between PIG and CG (for metabolism outcomes see Table 2).

Of the 26 (7 in CG, 6 ST, 13 ET) patients with diabetes at screening, there were 10 (1 in CG, 3 in ST, in 6 ET) in remission at 6 months. There were an additional 5 (1 in CG, 0 in ST, 4 in ET) who had improved to the status “prediabetes”. There were 8 (4 in CG, 2 in ST, 2 in ET) who retained their diabetes and there was a small amount of missing data which resulted in uncertain diabetes status (1 in CG, 1 in ST, 1 in ET).

### Lipid metabolism and inflammation parameters

There were no significant changes in the parameters of the lipid metabolism (cholesterol, triglycerides, HDL and LDL) and of inflammation (leukocytes, interleukin 6 and CRP) in comparison of the different groups (see Table 2).

### Quality of life

Valid data from IPAQ were available at screening from 80% (n = 74), at baseline in 91% (n = 81) of all patients, and at 6 months in 73% (n = 65) of all patients. The results include a physical activity category (low, moderate, high) and an estimate of the number of kcal expended per week based on the questionnaire and patients’ weight. There was an improvement in 6 months value from baseline of 2710 kcal/week (993 to 4428), but without significant superiority of any of the three groups (see Table 2).

A valid SF-36 could be obtained from ≥ 85% of the patients at baseline and at 6 months. Evaluation of the subscales physical functioning, physical role, body pain, general health, vitality, social functioning, emotional role, and mental health was performed. All subscales showed improvement but without significance differences between the groups. At baseline, the physical summary scores for the control, strength and endurance arms were 37 ± 9, 40 ± 10, 38 ± 10 respectively. At the 6-month follow-up, they were 49 ± 9, 51 ± 10, 50 ± 11. The corresponding mental summary scores were 48 ± 10, 49 ± 13, 51 ± 11 at baseline and 50 ± 10, 54 ± 9, 51 ± 10 at 6 months (see Table 2).

## Discussion

To the best of our knowledge, our study is the first controlled randomized trial comparing a structured strength training vs endurance training vs control group with normal activity regarding various outcome parameters after RYGB.

Our trial suggests that a structured postoperative training program after RYGB leads to significantly more TWL, EWL and fat loss but not fat-free mass, but these endpoints were not adjusted for multiple comparisons. Similar results are discussed in recent meta-analyses [31–33]. However, no differences were found between ET and ST. Our observed additional weight loss of + 2.5 kg in PIG compared to the control arm is in line with the literature for postoperative exercise programs. Interestingly, Mundbjerg et al. found a significant difference in TWL between the control and exercise group (combined aerobic and resistance training started 6 months postoperatively for 6.5 months) even after 24 months post intervention of 4.2 kg (95% CI: −0.2 to 8.3 kg; p = 0.042) [34].

As noted in the introduction, greater weight loss, especially in the first postoperative year, can lead to an improvement in the relapse rate of diabetes. Nonetheless, despite slightly greater weight loss, we saw no evidence in our primary endpoint for improved glucose concentrations after a 2 h-oGTT. It should be noted that only 28% of our patients had T2D and that 8.5% of them were insulin-dependent. It was therefore not possible to analyze the 2 h-oGTT in a diabetic subgroup. This may be particularly relevant since meta-analyses suggest that diabetics benefit most from a structured and supervised exercise program [19]. Furthermore, a predictive factor for diabetes hremission is shorter duration [35, 36], but unfortunately our collected data was not complete.

Other randomized trials of a structured exercise program after bariatric surgery also failed to find difference in fasting glucose [34, 37] or 2 h-oGTT [24], though their sample sizes were low and not powered for metabolic endpoints. Interestingly we did find some evidence for an activity-depending effect in our explorative sub-group analysis (active vs inactive), though the result was not significant. Other metabolic endpoints did not differ between PIG and control in our trial whereas Coen et al. [38] were able to show an effect on insulin sensitivity after 6 months of aerobic training after RYGB measured by intravenous glucose administration.

One reason for the lack of differences in our trial could be that the effect is masked by the massive weight loss (mainly in the first 6 months) and its positive metabolic consequences after RYGB. It is known that bariatric surgery alone can improve insulin sensitivity by up to 35% immediately after surgery [39]. Mundbjerg et al. was also able to show this in her study measured with the HOMA-index and single-point insulin sensitivity estimator (SPISE) 6 months after RYGB [34]. This masking effect may also apply to lipid metabolism, inflammation, and quality of life; this has also been shown in other studies [27]. However, if such masking effects exist, then the differences between groups must be small to remain undetected and their clinical relevance should be discussed.

Like other studies, we did not see a significant difference regarding prevention of the fat-free mass in the intervention groups, as an approximate correlate for muscle mass. A possible explanation for this result could be that the recommendations for protein intake are too low to rebuild muscle mass after RYGB (recommendations: protein intake of > 60 g/day or 1.5 g/kg ideal body weight per day [9, 40]); indications of this can be found in the BABS-study [41]. A prevention in fat-free mass after bariatric bypass surgery seems to correlate with a longer intervention period, as indicated by studies with 9–12 months of exercise training [31, 42] or a combined exercise training [43].

Several limitations in our trial design are noteworthy. A later start after the maximum weight loss and/or longer duration for the intervention may be more appropriate. An intravenous glucose measurement with a hyperglycemic clamp measurement would have been be interesting, but was not foreseen for logistical reasons and would probably not improve patient adherence. The choice of oGTT was problematic because of dumping symptoms, particularly at baseline, some weeks after surgery, though it was much less relevant at other points in time. oGTT-induced hypoglycaemia, especially after gastric bypass surgery, has also been described by other groups [44, 45]. Furthermore, it should also be noted that we did not include enough patients with diabetes mellitus type II to analyze the glucose metabolism of this subgroup. Finally, it should be noted here that in a randomized study, individual patient preferences with corresponding additional motivation for endurance or strength training cannot be accommodated, so that the patient’s metabolism cannot be addressed individually.

## Conclusion

Our study suggests that a postoperative, structured exercise program increases weight loss and fat loss. An effect in addition to gastric bypass surgery through endurance or strength training programs on glucose and lipid metabolism, inflammation or quality of life was not observed. There was no difference in any of the parameters measured between the endurance or strength training arm.

## Data Availability

No datasets were generated or analysed during the current study.
